# [Retracted] Acaricidal activity of extracts from *Ligularia virgaurea* against the *Sarcoptes scabiei* mite *in vitro*

**DOI:** 10.3892/etm.2026.13204

**Published:** 2026-06-08

**Authors:** Biao Luo, Fei Liao, Yanchun Hu, Xi Liu, Yajun He, Lei Wu, Hui Tan, Lijuan Luo, Yancheng Zhou, Quan Mo, Junliang Deng, Yahui Wei

Exp Ther Med 10:247–250, 2015; DOI: 10.3892/etm.2015.2503

Following the publication of the above paper, a concerned reader has drawn the Editor's attention to the fact that potential anomalies were identifiable within the two data panels in Fig. 1 on p. 249, including the apparent duplication of two pairs of the mites in Fig. 1B, albeit with some editing, resizing and/or rotation of the mites.

After having conducted an independent investigation of this paper in the Editorial Office, the Editor of *Experimental and Therapeutic Medicine* has determined that it should be retracted from the Journal on account of a lack of confidence in the authenticity of the presented data. The authors were asked for an explanation to account for these concerns, but the Editorial Office did not receive a satisfactory reply. The Editor regrets any inconvenience that has been caused to the readership of the Journal.

